# Understanding the needs of African researchers involved in implementation and dissemination science

**DOI:** 10.3332/ecancer.2025.1862

**Published:** 2025-03-04

**Authors:** Nwamaka Lasebikan, Catherine Oladoyinbo, Darby Walser, Ernest Kaninjing

**Affiliations:** 1 Oncology Center, University of Nigeria Teaching Hospital, Enugu 402109, Nigeria; 2 Federal University of Agriculture, Abeokuta 111101, Ogun State, Nigeria; 3 Georgia College & State University, Milledgeville, GA 31061, USA

**Keywords:** implementation science, research, implementation practice, Africa, capacity building

## Abstract

**Background:**

Implementation science focuses on the study and application of methods and strategies to facilitate the systematic adoption of evidence-based interventions and programs that enhance cancer prevention, detection, diagnosis, treatment and survivorship outcomes. Despite concerted efforts by African governments, international organizations and private sectors to mitigate the cancer burden, significant challenges persist in delivering effective and equitable cancer care across the continuum. The study assessed the needs of researchers and clinicians in Africa engaged in implementation and dissemination science.

**Methods:**

Data were gathered to explore gaps in collaboration, mentorship and funding while also examining the barriers and enablers to building implementation science capacity.

**Results:**

The findings revealed a growing interest in implementation science among African clinicians and researchers. However, significant challenges were identified, including difficulties in finding collaborators, limited access to mentors and inadequate funding opportunities. These gaps have hindered the ability of professionals to address the escalating cancer burden effectively.

**Conclusion:**

The study concluded that substantial gaps in implementation science capacity exist in Africa, which must be addressed to improve cancer care outcomes. Strengthening capacity in this field is critical to equipping researchers and clinicians to manage the rising cancer burden.

**Recommendations:**

The study recommends a multifaceted approach to building implementation science capacity in Africa. Key strategies include mentorship programs, targeted training, increased stakeholder involvement and the establishment of sustainable funding mechanisms to support research and practice in this area.

## Introduction

Implementation science involves the study and application of methods and strategies to promote the systematic uptake of evidence-based interventions and programs that can improve cancer prevention, detection, diagnosis, treatment and survivorship outcomes [1, 2]. Oncology implementation science is an emerging field that aims to bridge the gap between cancer research and its application in real-world settings [3]. In Africa, where the burden of cancer is increasing rapidly and health systems face significant challenges, building capacity in oncology implementation science is crucial for improving cancer care and outcome [4, 5]. Despite significant efforts made by African governments, international organizations and private sector to address the cancer burden in Africa, there are significant challenges in delivering effective and equitable cancer care along the entire cancer continuum. The landscape of implementation science in oncology in Africa is still in its early stages, with relatively limited research and funding in this field compared to other regions of the world [6]. However, there is growing interest and recognition of the importance of implementation science to improve cancer care and outcomes in Africa and the need for increased investment to support evidence-based interventions that can address the unique challenges of delivering cancer care in resource-limited settings. Success has also been linked to collaboration between researchers, healthcare providers, policymakers and patients to ensure that research findings are translated into actionable policies and practices that can improve cancer outcomes for patients in Africa.

Despite these challenges, there have been some notable initiatives in implementation science in oncology in Africa. For example, the African Organization for Research and Training in Cancer (AORTIC) hosted a workshop focused on implementation science during the 2021 AORTIC Virtual conference, to build capacity for cancer research and strengthen implementation practice in Africa, and facilitate the translation of research findings into policy and practice. At the end of the workshop, the implementation science special interest group was formed to advocate for better investment in training programs and capacity-building initiatives for oncology implementation science to ensure that there is a skilled workforce that can effectively translate research findings into practice and improve cancer care delivery in Africa.

To better understand the needs of the participants at the workshop, a needs assessment was conducted among participants of the implementation science conference to provide insights into the participants’ pathway from interest at the level of implementation experience, research and practice on the continent, as well as identify gaps and opportunities for future capacity building and inform the development of targeted interventions to improve capacity and capabilities for implementation science research and practice in Africa. In the African oncology landscape, implementation science is pivotal in optimizing cancer outcomes by identifying effective strategies for delivering cancer care within resource-constrained settings. Additionally, the translation of research findings into clinical practice is essential for enhancing the efficacy and accessibility of cancer care across the continent. By investing in oncology implementation science, we can address the current gaps and barriers to effective cancer care delivery and ultimately improve health outcomes for cancer patients in Africa. Therefore, the objective of this study is to conduct a needs assessment of African researchers and clinicians involved in implementation and dissemination science.

## Methods

A survey was employed to collect both qualitative and quantitative data. After IRB approval (IRB protocol number 17488 granted by the Georgia College Institutional Review Board), an e-mail introducing the study was sent to researchers and scientists across Africa who had attended the previous AORTIC conference on implementation science and had expressed an interest to be contacted in future research. An introductory e-mail came from an official AORTIC e-mail account and contained a link to take the survey. Only participants who provided consent were allowed to proceed and complete the survey electronically. The survey was available in English and French and data collection occurred in July and August 2022.

## Results

**Figure 1.**
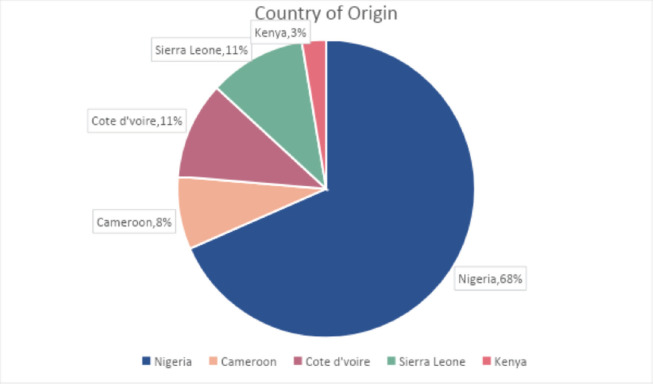
In which African country do you live or work?

**Figure 2.**
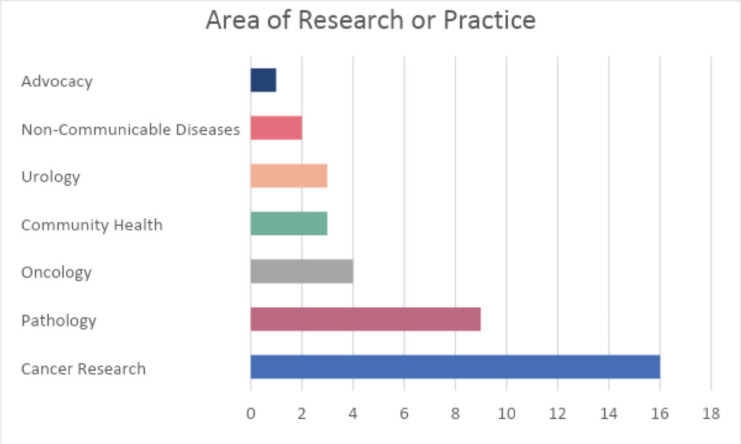
What is your area of research or practice?

**Figure 3.**
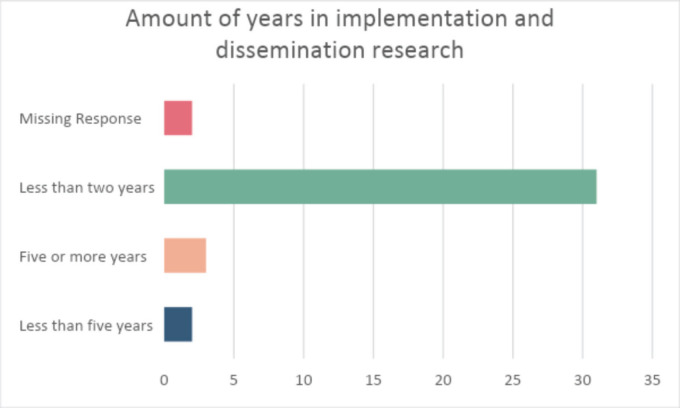
How many years have you been involved in implementation and dissemination science practice or research activities?

**Figure 4.**
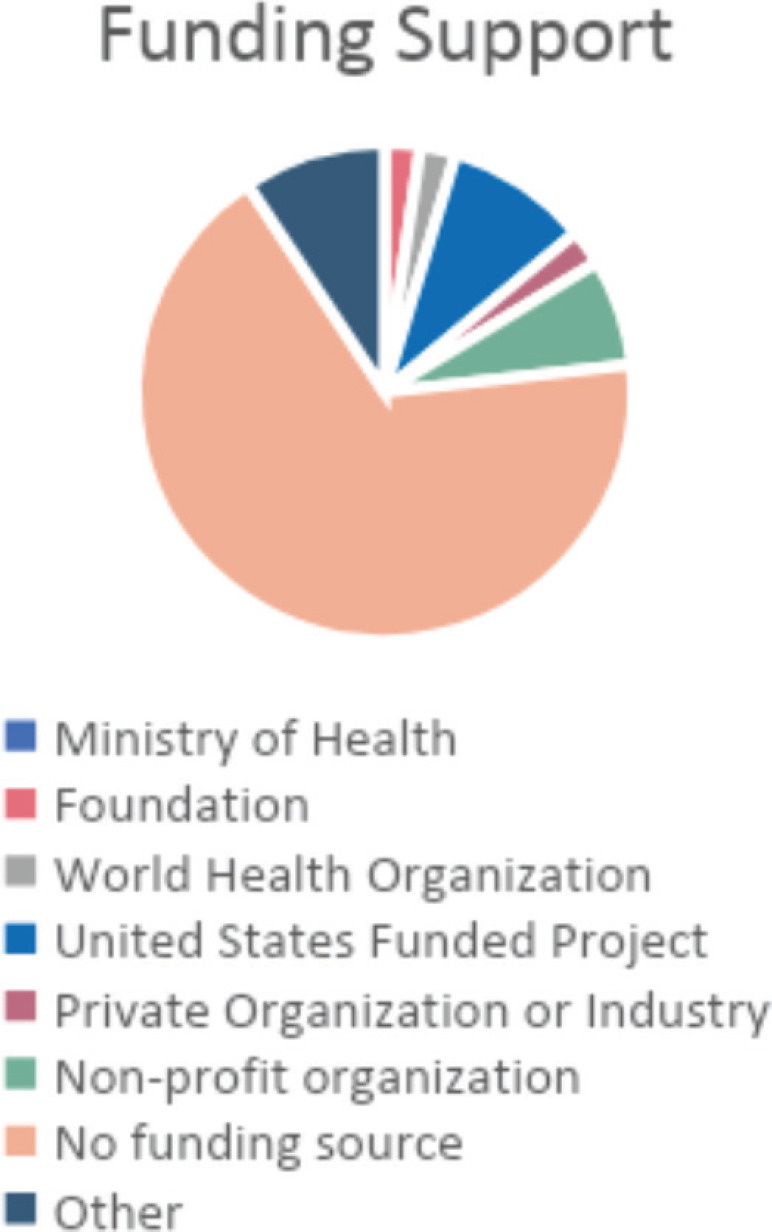
Which funding source(s) do you currently have to support your implementation and dissemination science activities?

**Figure 5.**
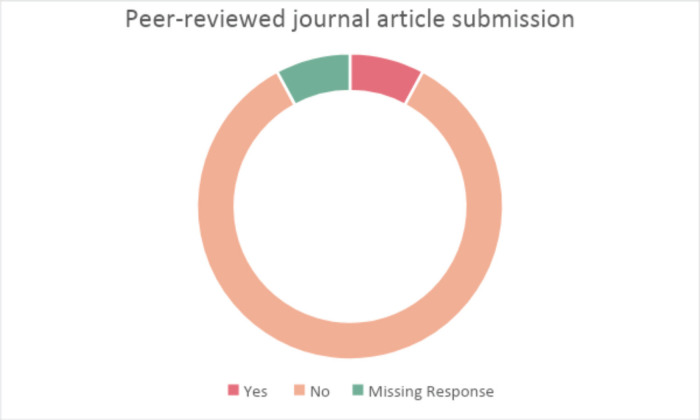
Have you published any peer-reviewed journal article(s) related to your activities in implementation and dissemination science?

A total of 139 potential participants received an e-mail with a link to take the survey and 38 completed the survey. Twenty-six (68%) participants were from Nigeria (see Figure 1), and 20 (52.6%) of participants were involved in both clinical work and research. Cancer research was the top area of research or practice for most of the study participants as shown in Figure 2. More than three quarters, 31 (81.5%) of participants reported that their involvement in implementation and dissemination research has been for less than 2 years (Figure 3), while 29

(67.4%) of participants indicated that they currently have no funding support for their implementation and dissemination science activities (Figure 4). Notably, 35 (92.1%) of participants indicated no published manuscript related to their implementation and dissemination science activities (Figure 5).

**Figure 6.**
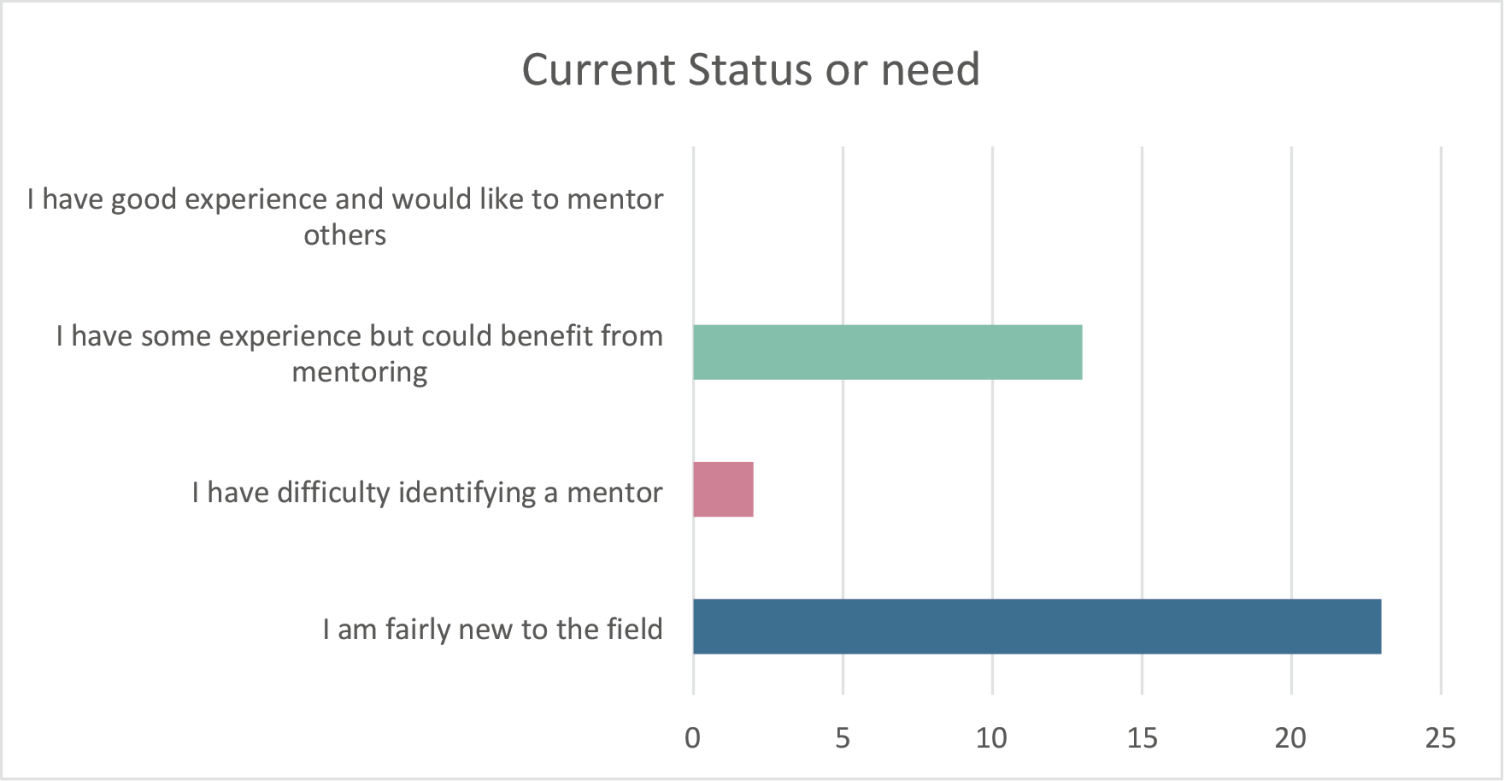
Which of the following statements best describes your current status or need?

**Figure 7.**
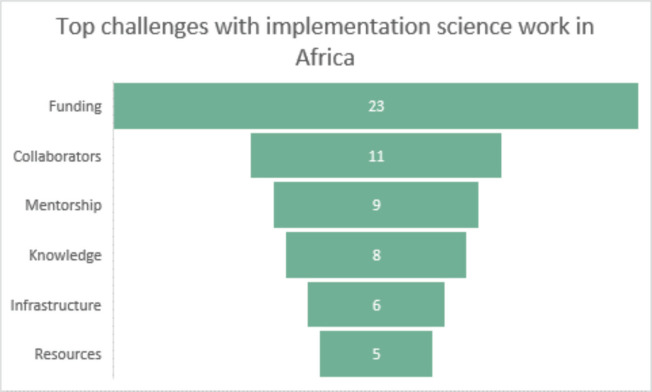
Please indicate the top three challenges or needs that individuals involved in implementation and dissemination science work in Africa face?

**Figure 8.**
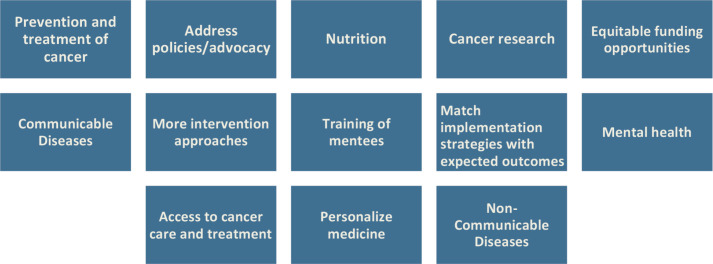
What are the main areas of opportunity for implementation and dissemination science that you see in Africa?

When asked to indicate the top three challenges or needs scientists and researchers in implementation science work face in Africa, funding, finding collaborators and mentoring were the most common obstacles raised (Figures 6 and 7). In terms of the top three areas of opportunity for implementation and dissemination science in Africa, participants listed prevention and treatment of cancer, policies/advocacy and nutrition (Figure 8). Participants also suggested that the Special Interest Group for Implementation and Dissemination Science in Africa should focus on cancer research, facilitating collaborations and training opportunities.

## Discussions

Implementation science seeks to promote the systematic uptake of evidence-based interventions and provides answers to bridge the gap ‘research to practice’ [7, 8]. The field has grown significantly over the years, especially in developed countries of the world, but this seems to be an emerging field in Africa [7, 9]. From this study, most of the respondents reported that they are relatively new to the science of implementation science, and lack of funding, collaborators and mentorship were identified as common barriers facing researchers in Africa. Ramaswamy *et al* [10] showed that no academic implementation science program exists in Africa outside of high-income countries. To build capacity in implementation and dissemination science, some approaches have been outlined in a systematic review by Davis and D’Lima [9] and include, teaching and training initiatives in the form of short courses, workshops and webinars [9, 11]. In a study by Semeere *et al* [12], only 9% of the respondents had previous training in implementation science, and previous training in implementation science was associated with three times the likelihood of reporting competence in behaviour change theory integration and framework use in intervention design and implementation.

When participants in the current study were asked about the top three areas of opportunity for implementation and dissemination science in Africa, participants listed prevention and treatment of cancer, policies/advocacy and nutrition among others. In a systematic review by Johnson *et al* [13], 53 articles were identified as articles that empirically evaluated or tested implementation strategies to improve cervical cancer prevention in Sub-Saharan Africa, out of which 16 (30.2%) were conducted in South Africa, 16 (30.2%) in Western, 14 (26.4%) in Eastern and 7 (13.2%) in Middle. The authors found no review that addresses implementation strategies to overcome sustaining cervical cancer prevention programs in SSA.

Iwelunmor *et al* [14] in another systematic review looked at the sustainability of health interventions implemented in Sub-Saharan Africa. The authors identified only 41 papers in the review and 26 countries were represented in the review, with Kenya and Nigeria having the most representation of available studies examining sustainability. With respect to policy/advocacy, only 4% of the research about policy implementation worldwide has been conducted in Africa [15]. Sturke *et al* [16] suggested that support for implementation science alone will have limited impact unless coupled with concerted efforts to bring researchers together with policymakers and program implementers.

## Conclusion

This study sought to examine the needs of researchers and clinicians in Africa involved in implementation and dissemination science. While there is a growing interest in this field, there are gaps in finding collaborators, mentors and funding that need to be addressed to ensure that clinicians and researchers in Africa are prepared to handle the growing burden of cancer on the continent. There is therefore a need to build implementation science capacity in Africa through mentorship, training, stakeholders’ involvement and adequate funding.

### Recommendations for advancing implementation science

Understanding the needs of local researchers and clinicians interested in implementation science holds significant opportunities for interventions to advance our cancer control efforts. For example, future research could focus on understanding and integrating local context-specific factors into the design and implementation of interventions, ensuring that interventions are culturally appropriate, feasible and effective within specific settings. Technology integration with the use of digital tools and technologies can facilitate training and capacity building. Increased funding and support for implementation research particularly in low- and middle-income countries can facilitate knowledge generation of locally relevant implementation tools for use in Africa.

## Study limitations

Our study has a number of limitations, including the size of the study participants as the response rate was 27%. Another significant limitation is the representation of study participants across Africa, and as such, the results may not be truly representative.

## Conflicts of interest

The authors declare no conflicts of interest.

## Funding

The authors have no funding declarations.

## References

[1] Cooper J, Murphy J, Woods C (2021). Barriers and facilitators to implementing community-based physical activity interventions: a qualitative systematic review. Int J Behav Nutr Phys Act.

[2] Marriott BR, Rodriguez AL, Landes SJ (2015). A methodology for enhancing implementation science proposals: comparison of face-to-face versus virtual workshops. Implement Sci.

[3] Practical guide to implementation science for surgical oncologists. https://link.springer.com/article/10.1245/s10434-021-10479-z.

[4] Cancer care workforce in Africa: perspectives from a global survey. https://infectagentscancer.biomedcentral.com/articles/10.1186/s13027-019-0227-8.

[5] Cancer health-care systems in Africa – the lancet. https://www.thelancet.com/series/cancer-health-care-systems-in-africa.

[6] Minding the gaps: implementation science research for improved cancer. https://criticalvalues.org/news/all/2021/12/20/minding-the-gaps-implementation-science-research-for-improved-cancer-care-in-africa.

[7] Rudd BN, Davis M (2020). Integrating implementation science in clinical research to maximize public health impact: a call for the reporting and alignment of implementation strategy use with implementation outcomes in clinical research. Implementation Sci.

[8] Ramaswamy R, Chirwa T, Salisbury K (2020). Developing a field of study in implementation science for the Africa region: the wits–UNC AIDS implementation science Fogarty D43. Pedagogy Health Promot.

[9] Davis R, D’Lima D (2020). Building capacity in dissemination and implementation science: a systematic review of the academic literature on teaching and training initiatives. Implementation Sci.

[10] Ramaswamy R, Mosnier J, Reed K (2019). Building capacity for Public Health 3.0: introducing implementation science into an MPH curriculum. Implementation Sci.

[11] Chambers DA, Proctor EK, Brownson RC (2017). Mapping training needs for dissemination and implementation research: lessons from a synthesis of existing D&I research training programs. Transl Behav Med.

[12] Semeere AS, Semitala FC, Lunkuse O (2021). An assessment of implementation science research capacity in Uganda. Health Res Policy Sys.

[13] Johnson LG, Armstrong A, Joyce CM (2018). Implementation strategies to improve cervical cancer prevention in sub-Saharan Africa: a systematic review. Implementation Sci.

[14] Iwelunmor J, Blackstone S, Veira D (2015). Toward the sustainability of health interventions implemented in sub-Saharan Africa: a systematic review and conceptual framework. Implementation Sci.

[15] Saetren H (2005). Facts and myths about research on public policy implementation: out-of-fashion, allegedly dead, but still very much alive and relevant. Policy Stud J.

[16] Sturke R, Vorkoper S, Bekker LG (2020). Fostering successful and sustainable collaborations to advance implementation science: the adolescent HIV prevention and treatment implementation science alliance. J Int AIDS Soc.

